# Phenotypic and genotypic diversity of airborne fungal spores in Demänovská Ice Cave (Low Tatras, Slovakia)

**DOI:** 10.1007/s10453-017-9491-5

**Published:** 2017-07-03

**Authors:** Rafał Ogórek, Bartosz Kozak, Zuzana Višňovská, Dana Tančinová

**Affiliations:** 1 Department of Genetics, Institute of Genetics and Microbiology, University of Wrocław, Przybyszewskiego Street 63/77, 51-148 Wrocław, Poland; 2 Department of Genetics, Plant Breeding and Seed Production, Wrocław University of Environmental and Life Sciences, Pl. Grunwaldzki 24a, 50-363 Wrocław, Poland; 3 Slovak Caves Administration, State Nature Conservancy of the Slovak Republic, Hodžova 11, 031-01 Liptovský Mikuláš, Slovakia; 4 Department of Microbiology, Faculty of Biotechnology and Food Sciences, Slovak University of Agriculture in Nitra, Tr. Hlinku 2, 949-76 Nitra, Slovakia

**Keywords:** Diversity, Culturable aeromycota, Fungal spores, Demänovská Ice Cave

## Abstract

This paper is the first aero-mycological report from Demänovská Ice Cave. Fungal spores were sampled from the internal and external air of the cave in June, 2014, using the impact method with a microbiological air sampler. Airborne fungi cultured on PDA medium were identified using a combination of classical phenotypic and molecular methods. Altogether, the presence of 18 different fungal spores, belonging to 3 phyla, 9 orders and 14 genera, was detected in the air of the cave. All of them were isolated from the indoor samples, and only 9 were obtained from the outdoor samples. Overall, airborne fungal spores belonging to the genus *Cladosporium* dominated in this study. However, the spores of *Trametes hirsuta* were most commonly found in the indoor air samples of the cave and the spores of *C. herbarum* in the outdoor air samples. On the other hand, the spores of *Alternaria abundans*, *Arthrinium kogelbergense*, *Cryptococcus curvatus*, *Discosia* sp., *Fomes fomentarius*, *Microdochium seminicola* and *T. hirsuta* were discovered for the first time in the air of natural and artificial underground sites. The external air of the cave contains more culturable airborne fungal spores (755 colony-forming units (CFU) per 1 m^3^ of air) than the internal air (from 47 to 273 CFU in 1 m^3^), and these levels of airborne spore concentration do not pose a threat to the health of tourists. Probably, the specific microclimate in the cave, including the constant presence of ice caps and low temperature, as well as the location and surrounding environment, contributes to the unique species composition of aeromycota and their spores in the cave. Thus, aero-mycological monitoring of underground sites seems to be very important for their ecosystems, and it may help reduce the risk of fungal infections in humans and other mammals that may arise in particular due to climate change.

## Introduction

The atmosphere is not a good environment for the life and development of microorganisms. Therefore, microorganisms are commonly present in this environment as airborne particles (spores and/or other propagation structures) suspended in the air, which form air fractions called bioaerosols (Kruczalak et al. 2002; Pusz et al. 2014; Ogórek et al. 2014a). Airborne particles affect visibility, climate, mammals’ health and the quality of life, because they are an important source of diseases for humans and animals (Lighthart 2000; Pavan and Manjunath 2014). The biological fraction of air can even represent half of air aerosol particles and with inhaled air can enter deep into the lungs (Pope et al. 1995; Srikanth et al. 2008). Moreover, the main component of bioaerosols is fungal spores, which are small and easily penetrate to the bronchi. This can result, e.g., in allergic respiratory diseases (Pekkanen et al. 2007; Żukiewicz-Sobczak 2013). However, it should be noted that sometimes such a large variety of fungi leads to difficulties in their identification to the species level, especially when only one way of identification is used, e.g., morphology or a single genetic marker. Additionally, many fungi reveal a different phenotype depending on the composition of the medium. Moreover, many fungi are closely related (Samson et al. 2011, 2014; Yilmaz et al. 2014; Visagie et al. 2014; Ogórek et al. 2016a).

Underground environments, like the air, are among the most inhospitable habitats for microbial life mainly due to low temperatures and lack of nutrients, especially for mycobiota (Poulson and Lavoie 2000; Pusz et al. 2015). Nevertheless, fungi and bacteria are very important groups of organisms for the ecology of underground sites, playing diverse roles in them, e.g., they can be decomposers or food for insects (Nováková 2009; Bastian et al. 2010; Vanderwolf et al. 2013). Overall, it should be noted that fungi mainly occur in the form of bioaerosols in underground environments, and they very rarely show visible growth, e.g., on rock surfaces as stains. Therefore, to a large extent, they are carried by air currents into the interior of the underground site from the external environment (Ogórek et al. 2014b, 2016b; Pusz et al. 2014). However, other factors also determine the presence of fungi in the underground sites, and the main ones include the season, flora outside the objects, air temperature and humidity, dripping and seeping water from the external environment, the number of visitors, the presence of animals (bats, arthropods) and their droppings (Mulec 2008; Ogórek et al. 2014a, 2016c; Griffin et al. 2014; Pusz et al. 2014, Kokurewicz et al. 2016).

Subterranean ecosystems can be a source of very specific and interesting microorganisms, including fungal extremophiles, which can either tolerate or are adapted to exploit unfavorable life conditions (Ogórek et al. 2017). It is commonly known that environmental stress is a major determinant of evolution. Overall, these microorganisms, in particular their enzymatic potentials, may be useful to industry, but they can also cause losses, e.g., in the food industry, and pose a biological threat to other living organisms including mammals (Niehaus et al. 1999; Nevo 2001; Eckburg et al. 2003; Johnson et al. 2013). On the other hand, it is important to mention that there has started to occur a temperature rise in the underground sites that will affect their ecosystems. Also, the progress of this process in underground sites depends in a large part on their location and depth (Domínguez-Villar et al. 2015). Moreover, it can be assumed that, as with other environments, the temperature rise in subterranean ecosystems can contribute, among other things, to changes in the fungal communities inhabiting them and can lead to new fungal diseases in mammals (Nadkarni and Solano 2002, Garcia-Solache and Casadevall 2010). For example, in the past several years, a new fungal disease has appeared in wintering bats, which was named white-nose syndrome (WNS). Generally, it is mainly associated with underground ecosystems, and its etiologic agent is the psychrophilic fungus *Pseudogymnoascus destructans*. WNS has already been discovered in many regions of the world, but it is the cause of mass mortality in hibernating bats mainly in the northeastern USA and Canada (Blehert et al. 2009; Gargas et al. 2009; Zukal et al. 2016). Many possible causes of WNS have been studied, and one hypothesis even suggested that the occurrence of this disease is associated with global warming. However, the current evidence does not support a relationship between climate change and WNS (Verant et al. 2012; Hoyt et al. 2015; Hayman et al. 2016). Nevertheless, it seems that aero-mycological and speleomycological research of underground sites related to climate change should be continued, because it can contribute to better understanding of the changes that occur in underground ecosystems and may help reduce the risk of new diseases associated with these places.

The present study aimed to (1) to assess the phenotypic and genotypic diversity of airborne fungal spores in Demänovská Ice Cave, (2) quantify their concentrations and (3) provide photographic documentation of colonial morphology and microscopic characteristics.

## Materials and methods

### Study area

The study was carried out in the Demänovská Ice Cave (49°01′62″N, 19°58′27″E). This cave is part of the biggest cave system in Slovakia, which is called the Demänovská Caves system (Piasecki et al. 2006). Its corridors are 1750 m long (tourist path 650 m), and the total difference in height is 57 m. The air temperature in permanent ice parts of the cave fluctuates around 0 °C, and it rises to 5.7 °C near the entrance. The air relative humidity is between 92 and 98% (Slovak Caves Administration 2016). Demänovská Ice Cave is a very popular attraction in Slovakia. It is estimated that 3,133,416 tourists visited the caves from 1970 to 2014 and in the year of this research 70,769 tourists (Nudziková 2014).

### Sampling methods

**Fig. 1:**
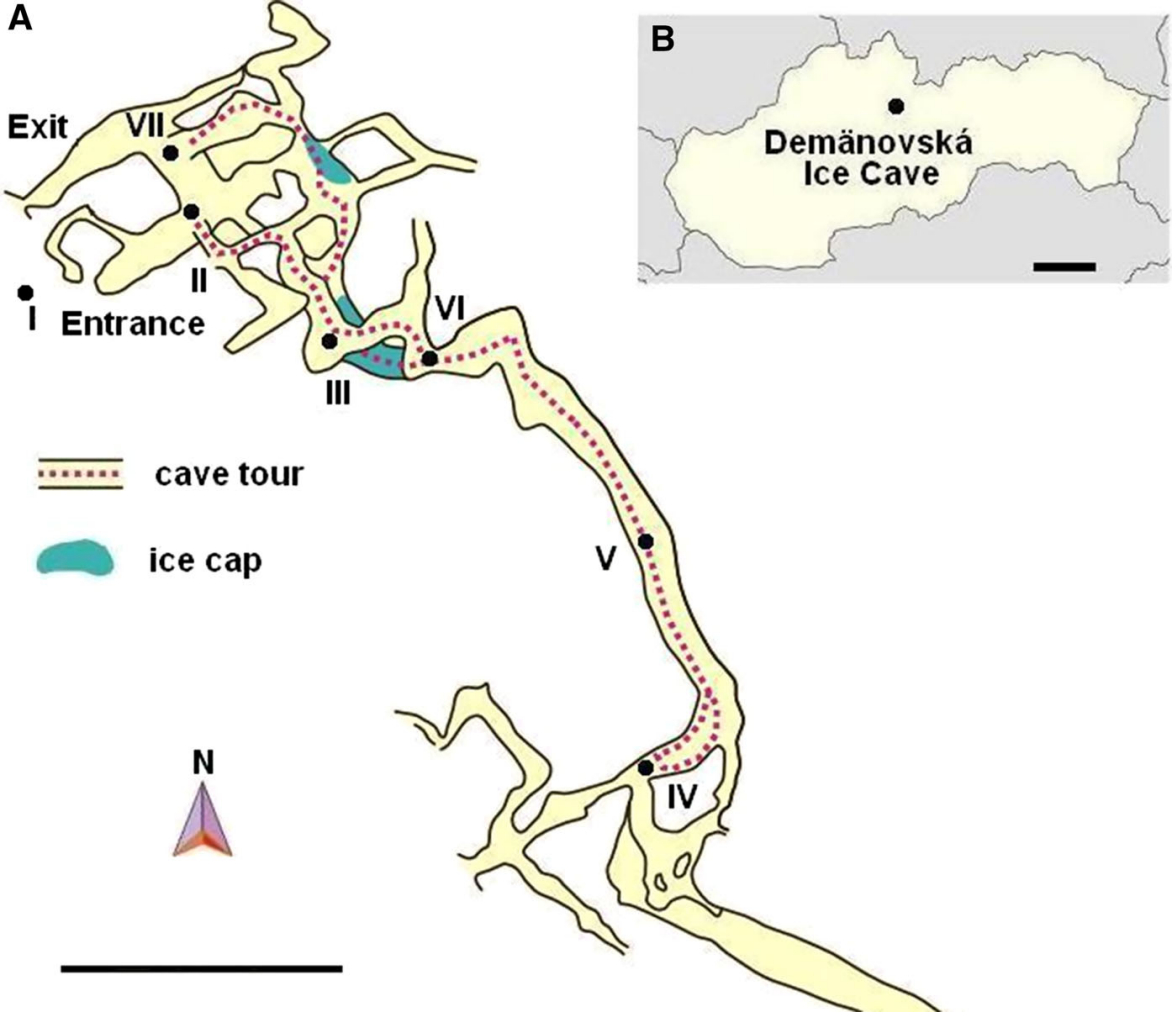
Demänovská Ice Cave in Slovakia:  **A** map and outline of the tourist route, **B** geographic location. Fungal sampling points: *I* from 3 to 4 m in front of the entrance of the cave, and from *II* to *VIII* inside of it. *Scale bars A* = 100 m, *B* = 50 km

The air samples for mycological studies were collected on the morning of June 5, 2014, according to the method described by Ogórek et al. (2013) with the sampler “Air Ideal 3P” (bioMérieux) and potato dextrose agar medium (PDA, Biocorp) (Fig. 1).

### Phenotypic and molecular identification of fungi

**Fig. 2:**
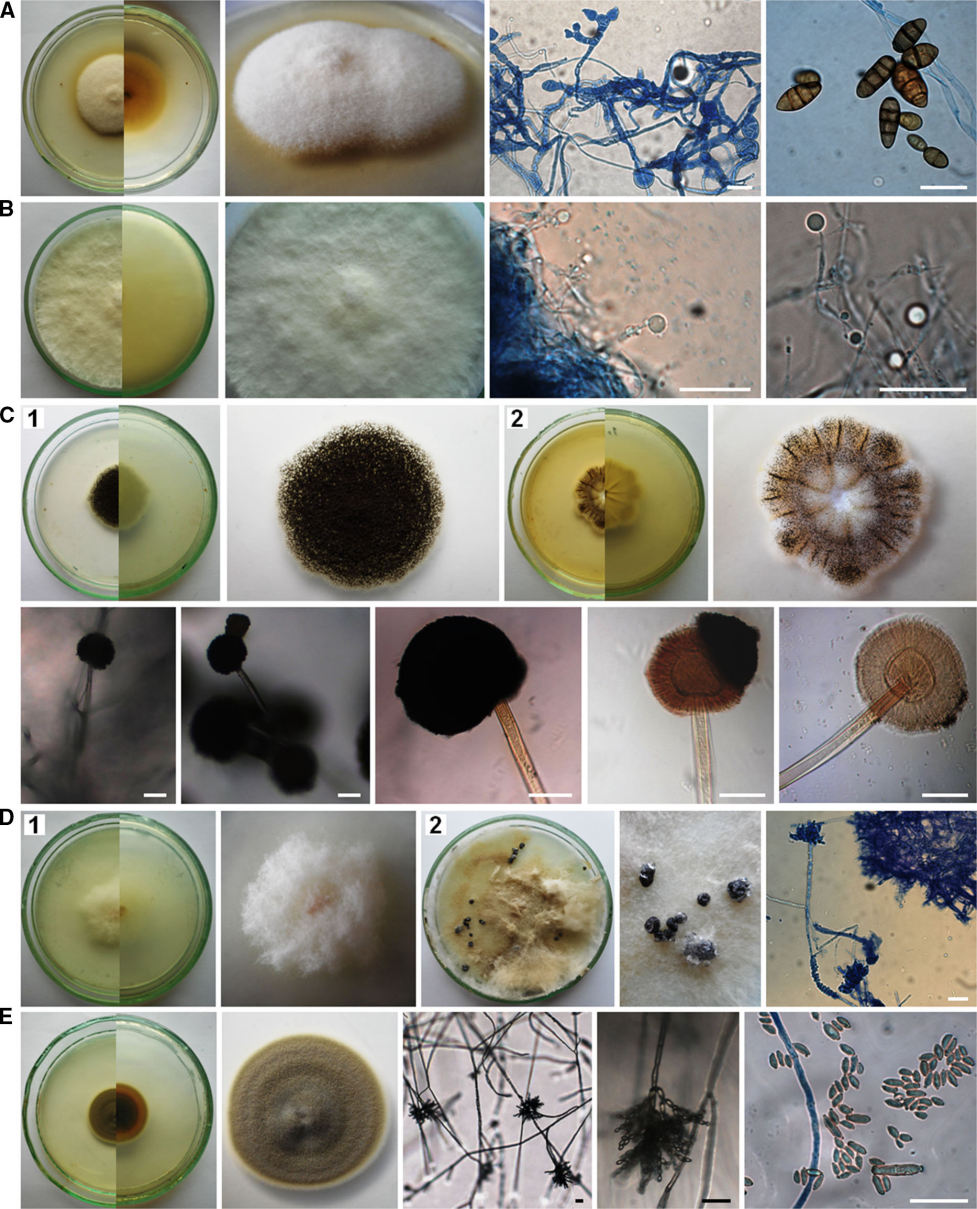

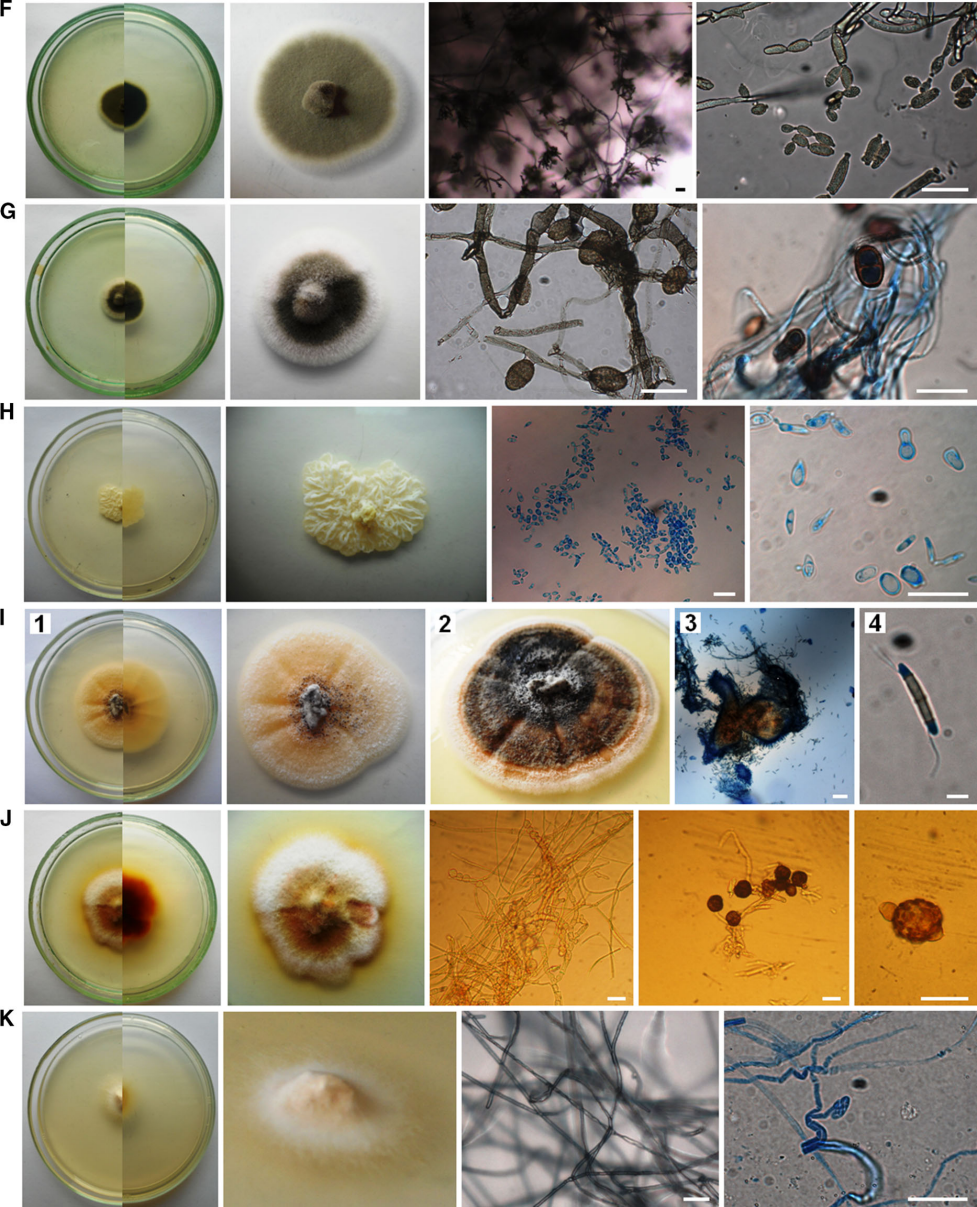

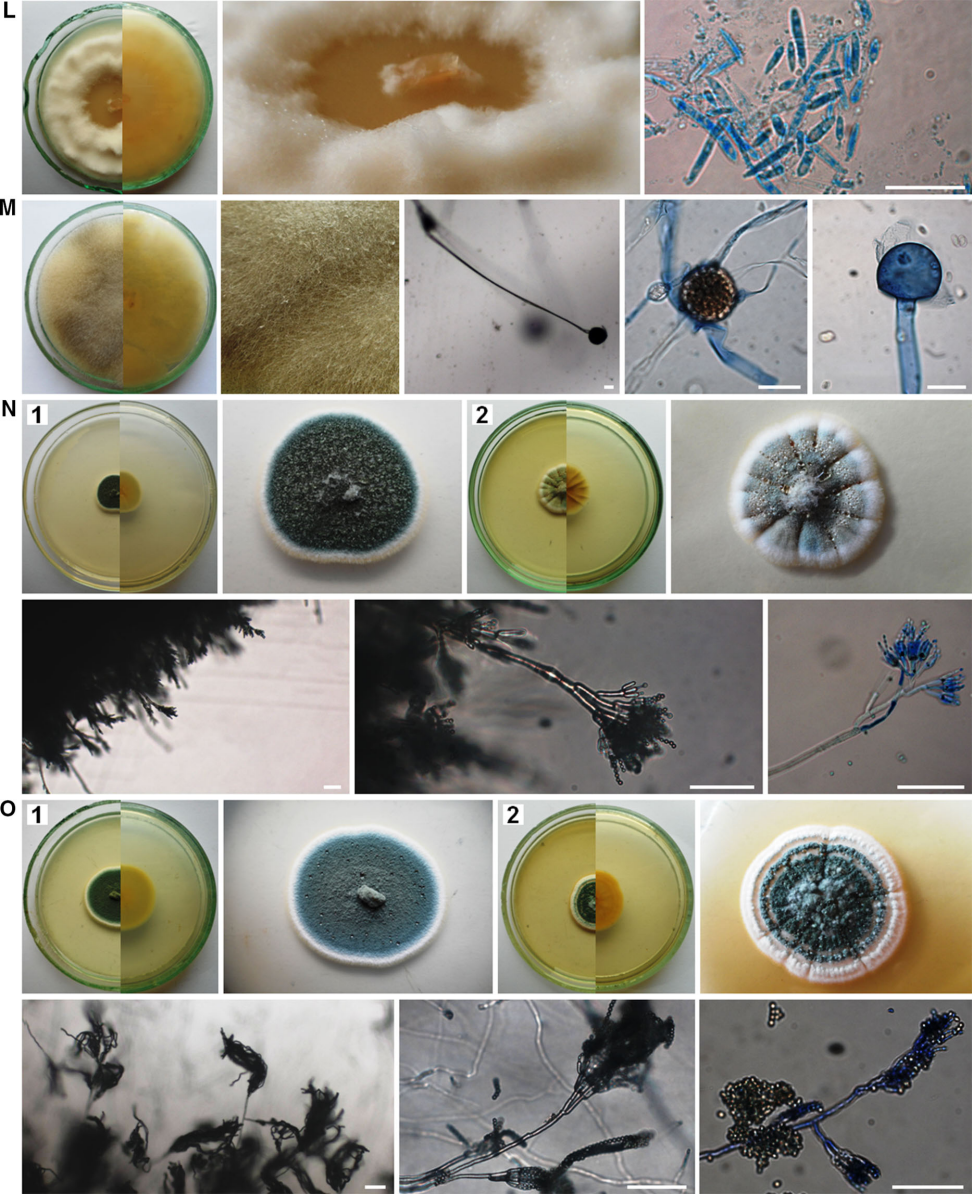

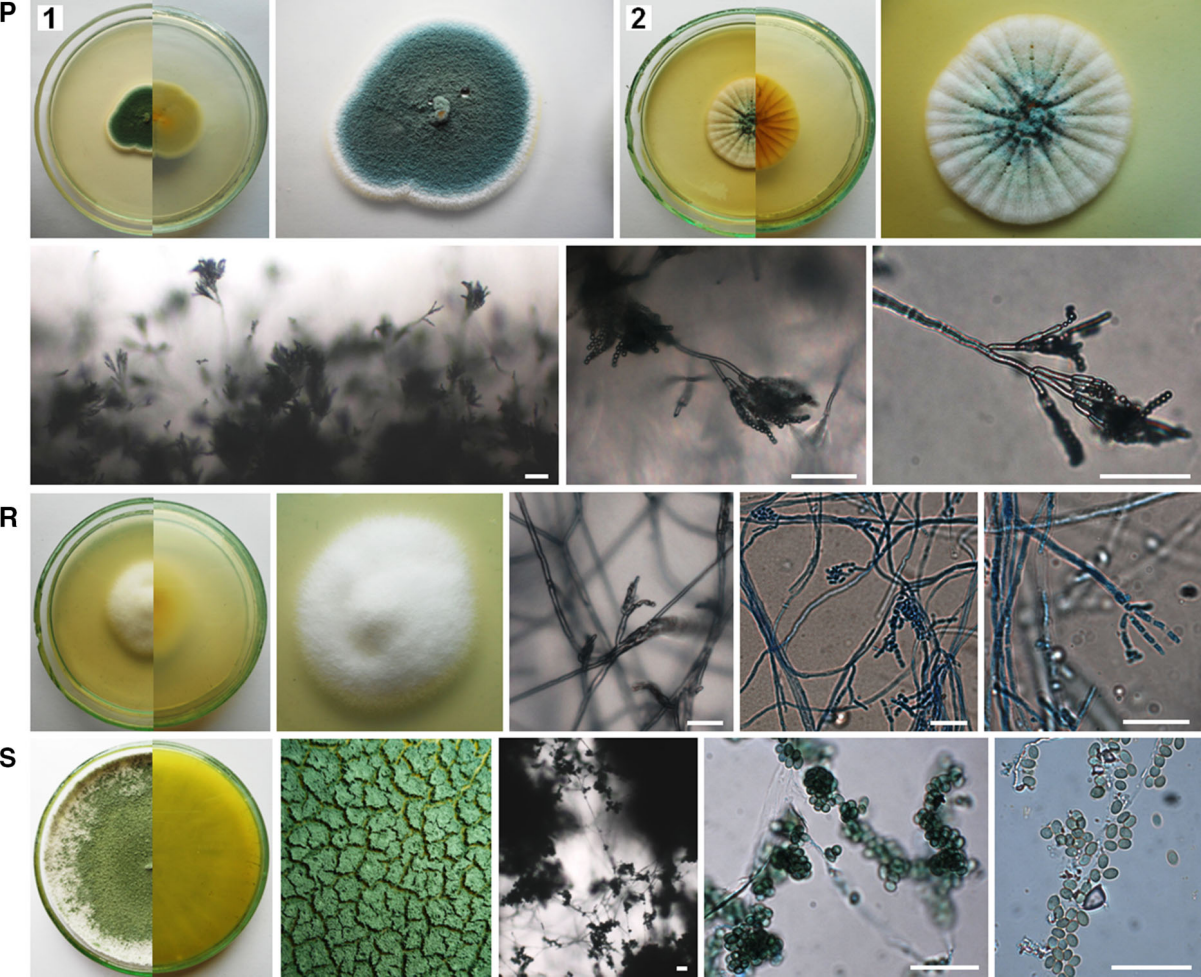
Culturable aeromycota of the Demänovská Ice Cave, 7-day-old or 21-day-old culture (**D2**, **I2**) at 25 ± 1 °C, *top* and *bottom* view of a colony on PDA and CYA media (**C2**, **N2**, **O2**, **P2**) and the characteristic structure of fungi under the optical microscope on PDA medium: **A** *Alternaria abundans*, **B** *Arthrinium kogelbergense*, **C** *Aspergillus niger*, **D** *Botrytis cinerea*, **E** *Cladosporium cladosporioides*, **F** *C. herbarum*, **G** *C. macrocarpum*, **H** *Cryptococcus curvatus*, **I** *Discosia* sp., **J** *Epicoccum nigrum*, **K** *Fomes fomentarius*, **L** *Microdochium seminicola*, **M** *Mucor hiemalis*, **N** *Penicillium brevicompactum*, **O** *P. commune*, **P** *P. crustosum*, **R** *Trametes hirsuta*, **S** *Trichoderma longibrachiatum*. *Scale bars* *I4* = 5 µm; *A*, *B*, *D*–*H*, *K*–*M*, *R*, *S* = 20 µm; *C*, *I3*, *N*–*P* = 50 µm

The air samples in Petri dishes with PDA were incubated from 4 to 21 days at 25 ± 1 °C. Then, the colonies that appeared on the medium were counted and the fungi were identified using the taxonomic literature (Ellis 1971; Pitt 1979; Pitt and Hocking 2009; Watanabe 2011; Samson et al. 2011, 2014; Visagie et al. 2014; Yilmaz et al. 2014) and genetic analysis. Generally, macro- and micromorphological features of fungi were observed on PDA medium and additionally on Czapek yeast autolysate agar (CYA, Pitt 1979) in the case of *Penicillium* and *Aspergillus* spp. (Fig. 2).

DNA from fungi cultured on PDA was isolated according to the method described by Doyle and Doyle (1987) modified by Ogórek et al. (2012). The internal transcribed spacer region of fungal rDNA was amplified using the primers ITS1 (5′-TCCGTAGGTGAACCTGCGG-3′) and ITS4 (5′-TCCTCCGCTTATTGATATGC-3′) (White et al. 1990). Polymerase chain reactions were performed in a T100 Thermal Cycler (Bio-Rad) according to Ogórek et al. (2016a). The PCR products were verified by electrophoretic separation on 1.2% agarose gel, purified using Clean-UP (A&A Biotechnology) and sequenced by Macrogen Europe (Netherlands).

### Data analyses

BioEdit Sequence Alignment Editor was used for the analysis of the obtained fungal ITS sequences (http://www.mbio.ncsu.edu/bioedit/bioedit.html). Then, fungi were identified to the species level using the BLAST algorithm (http://www.ncbi.nlm.nih.gov/) that compared the obtained sequences with those deposited in the GenBank database. The sequences obtained during the study were also placed in the GenBank databases (Table 1), and they were used to create the neighbor-joining phylogenetic tree with 1000 bootstrap replicates (Saitou and Nei 1987). Evolutionary analyses were conducted in MEGA7, and the maximum composite likelihood method was used to determine the evolutionary distances (Tamura et al. 2004; Kumar et al. 2016).

**Table 1:** Culturable aeromycota of the Demänovská Ice Cave and BLAST analysis

Species	Air		Temple	Query cover (%)	Identities (%)	*E* value	GenBank accession numbers
	Outdoor	Indoor					
*Alternaria abundans* (E.G. Simmons) Woudenberg & Crous		+	KF876825.1	99	99	0.0	KX426947.1
*Arthrinium kogelbergense* Crous	+	+	KF144895.1	100	99	0.0	KX426951.1
*Aspergillus niger* Tiegh.	+	+	KP940595.1	100	99	0.0	KX426952.1
*Botrytis cinerea* Pers.	+	+	KJ476698.1	97	99	0.0	KX426945.1
*Cladosporium cladosporioides* (Fresen.) G.A. de Vries	+	+	HM776418.1	99	99	0.0	KX426943.1
*Cladosporium herbarum* (Pers.) Link	+	+	KP151612.1	100	99	0.0	KX426942.1
*Cladosporium macrocarpum* Preuss 1848		+	KU925906.1	99	99	0.0	KX426953.1
*Cryptococcus curvatus* (Diddens & Lodder) Golubev		+	EU266558.1	97	97	0.0	KX426955.1
*Discosia* sp.		+	KU325418.1	86	96	0.0	KX426948.1
*Epicoccum nigrum* Link		+	KR094452.1	100	100	0.0	KX426949.1
*Fomes fomentarius* (L.) Fr.	+	+	FJ865441.1	98	99	0.0	KX426954.1
*Microdochium seminicola* M. Hern.-Restr., Seifert, Clear & B. Dorn		+	KP859021.1	100	99	0.0	KX426950.1
*Mucor hiemalis* Wehmer		+	JQ683255.1	100	99	0.0	KX426958.1
*Penicillium brevicompactum* Dierckx	+	+	KP942935.1	98	99	0.0	KX426946.1
*Penicillium commune* Thom		+	AF455471.1	97	91	0.0	KX426957.1
*Penicillium crustosum* Thom	+	+	HM037943.1	91	89	0.0	KX426959.1
*Trametes hirsuta* (Wulfen) Lloyd	+	+	KC920740.1	100	100	0.0	KX426944.1
*Trichoderma longibrachiatum* Rifai		+	JF694937.1	99	99	0.0	KX426956.1
*∑* species	9	18	–				

A + indicates that the fungus was cultured from the samples

The obtained data from the number of airborne fungal colonies cultured in Petri dishes were expressed as the CFU (colony-forming units) per cubic meter of air (CFU m^−3^) using the formula: *X* = (*a* × 1000)/*V*, where “*a*” is the number of colonies obtained on a Petri dish, and “*V*” is the air volume sampled (m^3^). Then, the data were subjected to statistical analysis using the Statistica 12.0 package. For this purpose, one-way analysis of variance (ANOVA) and the Tukey HSD (honest significant differences) test were used. The level of significance was set at *α* ≤ 0.05.

## Results

Altogether, the presence of eighteen different culturable airborne fungi spores was determined in the speleomycology research of the Demänovská Ice Cave. All of them were isolated from the indoor samples (17 filamentous fungi and 1 yeast-like fungus), and only 9 filamentous fungi were obtained from the outdoor samples. The species *Alternaria abundans*, *Cladosporium macrocarpum*, *Cryptococcus curvatus*, *Discosia* sp., *Epicoccum nigrum*, *Microdochium seminicola*, *Mucor hiemalis*, *Penicillium commune* and *Trichoderma longibrachiatum* were discovered only in the air inside the cave (Table 1). However, the highest concentration of culturable airborne fungal spores was found in the outdoor air samples (755 CFU m^−3^); *p*_I,II_ < 0.001, *R* ^2^ = 11.02%. On the other hand, the indoor air samples contained from 47 to 273 fungal CFU in 1 m^3^, and the highest spore concentrations were recorded for the second location; the smallest for the locations from III to VI; *p*_II,*V*_ < 0.001, *R* ^2^ = 11.02% (Table 2; Fig. 1).

**Table 2:** Average number of culturable fungal spores of the Demänovská Ice Cave (CFU per m^3^ of air)

Sampling location	Species	CFU·m^−3^		
		Species		In total
I^a^	*Arthrinium kogelbergense*	6.7	c^b^	755.0 A^b^
	*Aspergillus niger*	3.3	c	
	*Botrytis cinerea*	46.7	bc	
	*Cladosporium cladosporioides*	146.7	b	
	*Cladosporium herbarum*	493.3	a	
	*Fomes fomentarius*	20.0	bc	
	*Penicillium brevicompactum*	10.0	c	
	*Penicillium crustosum*	13.3	c	
	*Trametes hirsuta*	15.0	c	
II	*Alternaria abundans*	15.0	b^b^	273.0 B^b^
	*Arthrinium kogelbergense*	10.0	b	
	*Aspergillus niger*	5.0	b	
	*Botrytis cinerea*	11.6	b	
	*Cladosporium cladosporioides*	88.2	a	
	*Cladosporium herbarum*	90.0	a	
	*Cryptococcus curvatus*	3.3	b	
	*Discosia* sp.	6.6	b	
	*Epicoccum nigrum*	13.3	b	
	*Microdochium seminicola*	1.7	b	
	*Trametes hirsuta*	28.3	b	
III	*Botrytis cinerea*	5.1	a^b^	47.0 D^b^
	*Cladosporium herbarum*	11.7	a	
	*Cladosporium macrocarpum*	3.3	a	
	*Mucor hiemalis*	6.7	a	
	*Penicillium brevicompactum*	8.3	a	
	*Penicillium crustosum*	1.8	a	
	*Trametes hirsuta*	10.1	a	
IV	*Aspergillus niger*	11.7	a^b^	50.0 D^b^
	*Botrytis cinerea*	18.3	a	
	*Penicillium commune*	13.3	a	
	*Trichoderma longibrachiatum*	6.7	a	
V	*Aspergillus niger*	46.7	a^b^	75.0 D^2^
	*Penicillium brevicompactum*	6.7	b	
	*Penicillium commune*	10.0	b	
	*Trametes hirsuta*	11.6	b	
VI	*Fomes fomentarius*	16.7	ab^b^	72.0 D^b^
	*Mucor hiemalis*	1.7	b	
	*Penicillium brevicompactum*	10.0	ab	
	*Penicillium commune*	15.0	ab	
	*Trametes hirsuta*	28.6	a	
VII	*Alternaria abundans*	10.0	b^b^	198.3 C^b^
	*Arthrinium kogelbergense*	11.7	b	
	*Aspergillus niger*	15.0	b	
	*Botrytis cinerea*	56.7	a	
	*Cladosporium cladosporioides*	8.3	b	
	*Cladosporium herbarum*	4.9	b	
	*Fomes fomentarius*	5.0	b	
	*Penicillium brevicompactum*	6.7	b	
	*Trametes hirsuta*	80.0	a	

^a^I—the outdoor air samples, II–VII—the indoor air samples

^b^For each location, the numbers of fungal spores followed by the same letter are not statistically different, and others are (Tukey HSD test, *α* ≤ 0.05). Small letters indicate the differences between fungal species in a given location. Capital letters indicate the effect of a particular location on the total concentration of fungal spores

**Fig. 3:**
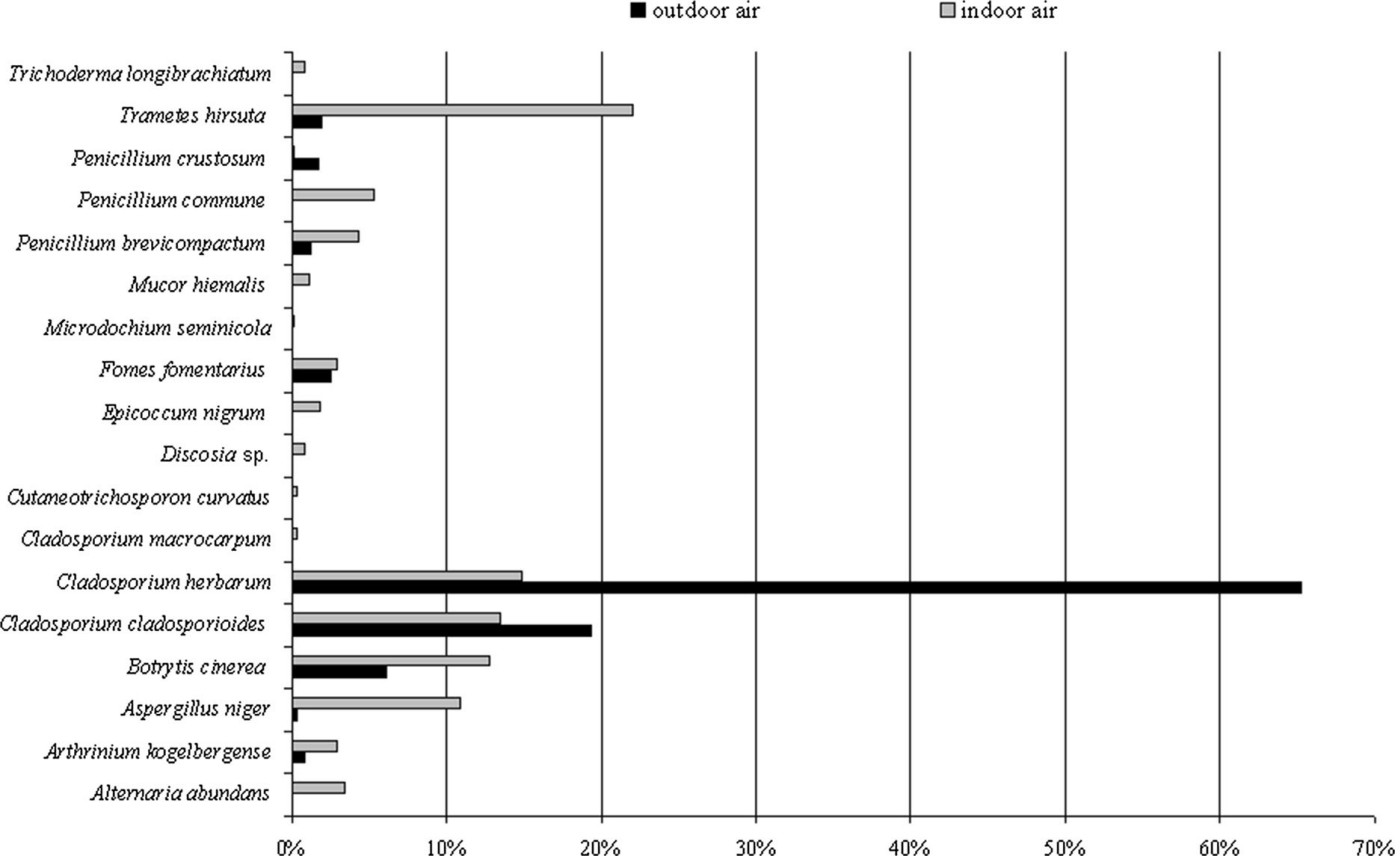
The percentage of each species of culturable aeromycota isolated contributing to the totals for the outdoor and indoor air samples in the Demänovská Ice Cave

Overall, fungal spores belonging to *Cladosporium* genera dominated in the air of the cave. However, the spores of the particular species *Trametes hirsuta* were most commonly discovered in the indoor air samples of Demänovská Ice Cave (in five of six tested locations), and they constituted approximately 22% of all found airborne fungal spores. On the other hand, fungal spores belonging to *C. curvatus*, *Discosia* sp., *Epicoccum nigrum*, *Microdochium seminicola*, *Penicillium crustosum* and *Trichoderma longibrachiatum* were much less commonly found in the indoor air (only in one of six tested locations), and they constituted 1.8% of all fungal spores (Table 2; Figs. 2, 3). Generally, the spores of *C. herbarum* were most commonly found in the outdoor air samples (*p*_*C. herbarum*, *C. cladosporioides*_ < 0.001, *R* ^2^ = 84.93%), and they constituted approximately 65% all fungal spores. On the other hand, the spores of fungi belonging to *Arthrinium kogelbergense*, *Aspergillus niger*, *P. brevicompactum*, *P. crustosum* and *Trametes hirsuta* were the least common in the same air samples (*p*_*T. hirsuta*, *C. cladosporioides*_ <0.001, *R* ^2^ = 84.93%), and they constituted 2% of all fungal spores (Table 2; Figs. 2, 3). However, the species composition of the fungal spores in the indoor air samples depended on the sampling site. The spores of *C. cladosporioides* and *C. herbarum* were most commonly discovered in the second location (*p*_*C. cladosporioides*_, _*T. hirsuta*_ and *p*_*C. herbarum*, *T. hirsuta*_ < 0.001, *R* ^2^ = 59.72%), *A. niger* in the fifth location (*p*_*A. niger*, *T. hirsuta*_ = 0.035, *R* ^2^ = 43.05%), *T. hirsuta* in the sixth location (*p*_*T. hirsuta*, *M. hiemalis*_ = 0.037, *R* ^2^ = 28.81%), and *Botrytis cinerea* and *T. hirsuta* (*p* > 0.050, *R* ^2^ = 64.43%) in the seventh location (*p*_*B. cinerea*__,__*A. niger*_ = 0.014 and *p*_*T. hirsuta*, *A. niger*_ <0.001, *R* ^2^ = 64.43%). In the case of the third and fourth locations, all airborne fungal spores were isolated at the same level—there were no significant differences (Table 2; Figs. 1, 2, 3).

**Fig. 4:**
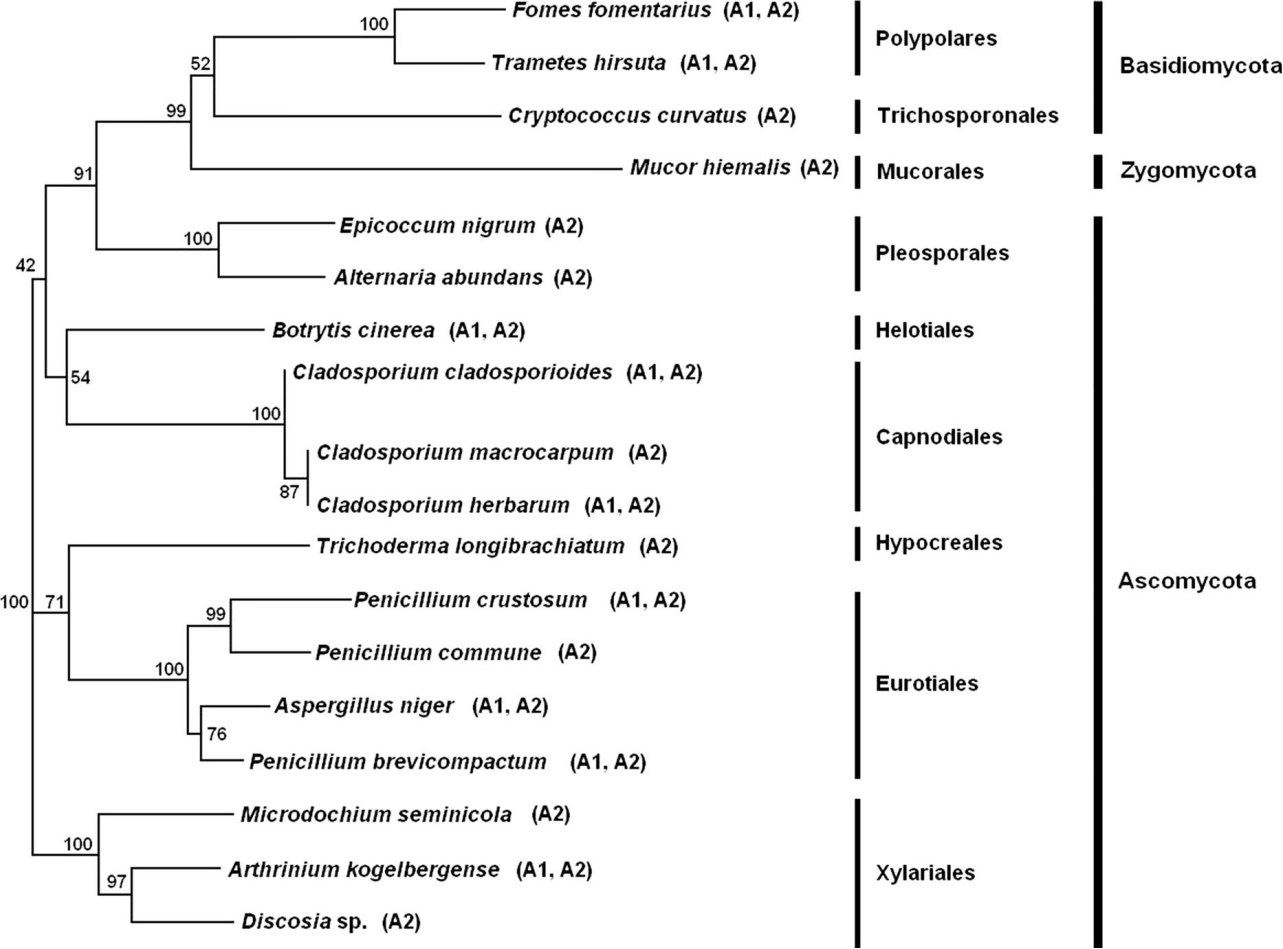
Phylogenetic tree based on ITS sequence of culturable aeromycota in the Demänovská Ice Cave showing the relationship of species isolated from the outdoor samples (*A1*) and the indoor samples (*A2*)

The neighbor-joining phylogenetic tree based on ITS1 and 4 rDNA gene sequences obtained from 18 fungal species belonging to 3 phyla and 9 orders includes three main clusters. The first cluster contains only three species, all belonging to the order Xylariales (Ascomycota), and they are divided into two subclusters: the first with *Discosia* sp. and *A. kogelbergense* and the second with *M. seminicola*. The second main cluster contains five species, which also belong to the phylum Ascomycota and are divided into two main subclusters: four species of fungi, which all belong to the order Eurotiales (*P. brevicompactum*, *A. niger*, *P. commune*, *P. crustosum*), and only *T. longibrachiatum* from Hypocreales. The third cluster is formed by the largest group of fungal species, which belong to three phyla and six orders. Species such as *B. cinerea* (Helotiales), *C. cladosporioides*, *C. herbarum* and *C. macrocarpum* (Capnodiales) from the phylum Ascomycota form the first main subcluster. The second subcluster contains two species belonging to the phylum Basidiomycota and the order Polyporales (*Fomes fomentarius*, *T. hirsuta*); one species belongs to the phylum Basidiomycota and order Trichosporonales (*Cryptococcus curvatus*), another species belongs to the phylum Zygomycota and order Mucorales (*Mucor hiemalis*), and two species belong to the phylum Ascomycota and order Pleosporales (*Alternaria abundans*, *E. nigrum*) (Fig. 4).

## Discussion

The phylum Ascomycota dominates the fungal community in ecosystems of caves and mine caves worldwide, and they constitute approximately 69% of all cultured fungi—Basidiomycota (20.0%), Zygomycota (6.6%), Mycetozoa (2.6%), Oomycota (1.0%), and 0.8% other (Vanderwolf et al. 2013). Generally, our results agree with the above reports, because we cultured from the air in the Demänovská Ice Cave ca. 77.8% of airborne fungi species from Ascomycota, 16.7% from Basidiomycota, and 5.5% from Zygomycota. Particularly noteworthy are the species of Basidiomycota, because these fungi grow slowly in vitro (Vanderwolf et al. 2013). On the other hand, currently approximately 39 genera of fungal spores have been detected in the air of Slovakian caves (Nováková 2009; Ogórek et al. 2016a, b, c, d). The air samples of the Demänovská Ice Cave contained 9 genera among them. Additionally, we have discovered five new genera for the bioaerosols of caves worldwide: *Cryptococcus*, *Discosia*, *Fomes*, *Microdochium* and *Trametes*.

Moreover, we detected fungal spores belonging to 18 species in the inside air of the Demänovská Ice Cave. Most of them are commonly found in the bioaerosols of caves worldwide (Nováková 2009; Vanderwolf et al. 2013). However, *Alternaria abundans*, *Arthrinium kogelbergense*, *Cryptococcus curvatus*, *Discosia* sp., *Fomes fomentarius*, *Microdochium seminicola* and *Trametes hirsuta* spores were discovered in the environment of natural and artificial underground sites for the first time worldwide, especially in the air. *F. fomentarius* and *T. hirsute* were previously isolated from wood in mines, but never before from the air of underground ecosystems (Vanderwolf et al. 2013). It should also be emphasized that most of those fungi can be identified only to a section, complex or genus level, when only morphological analysis or a single genetic marker is used, because their phenotypes and genotypes are similar to each other (Schoch et al. 2012). Therefore, we used a combination of classical phenotypic and molecular methods to identify these fungi to the species level.

We decided to carry out research in July, because most cultivable fungal spores are present in the air during the summer (Wang et al. 2010). Generally, during this period, the fungal spores belonging to *Cladosporium* are commonly found in the air of Europe, as well as in the air of Slovakian caves (Stepalska et al. 1999; Ogórek et al. 2016b, d; Sadyś 2017). This is also confirmed by our results, but, given the particular species *C. herbarum*, spores were most detected in the outdoor samples of the cave, and *T. hirsuta* spores in the case of the indoor air of the cave. It should be emphasized that airborne fungal spores can be very dangerous to mammal health; e.g., they are strongly associated with allergic respiratory diseases, especially asthma (Pekkanen et al. 2007). However, the biological threat depends on the species of fungus spores to which they belong as well as their concentration. In the case of *T. hirsuta*, it grows easily on wood of hardwood and conifer trees, and it does not endanger the health of mammals (Kuhad et al. 1997). Probably, one reason for the occurrence of large numbers of *T. hirsuta* spores in the cave is its growth on the wood stumps deposited by people in the cave, e.g., when creating a tourist route. On the other hand, the above-mentioned *Cladosporium* spores (*C. cladosporioides* and *C. herbarum*) are highly allergenic, but a minimum of 2800 airborne spores in 1 m^3^ present a biological hazard associated with respiratory allergies in humans (Rapiejko et al. 2004). Thus, the levels of *Cladosporium* spores in the air of the Demänovská Ice Cave did not pose a biological hazard to humans.

In the literature, it is also reported that the overall concentration of culturable airborne fungal spores inside caves during summer is lower than in the external environment, but aeromycota occurring in the underground sites is richer in species (Pusz et al. 2014; Ogórek et al. 2014b). This is confirmed by the results of these studies. However, it should be emphasized that the numbers of culturable fungal spores detected in the external and internal air of the Demänovská Ice Cave were at much lower levels than in other Slovakian caves (Ogórek et al. 2016b, c, d). Moreover, they did not exceed the limit of fungal air quality, which, e.g., according to the American Industrial Hygiene Association should not be higher than 1000 airborne spores per m^3^ (Choi et al. 1999). This low concentration level of fungal spores may be related to the specific microclimate in the cave such as the mentioned low temperature in the cave (from 0 to 5.7 °C) and thus the constant presence of ice caps in the cave (Fig. 1). Other factors may include the location of the cave, which is surrounded by mountain peaks, as well as the elevation, or the flora composition. These reasons directly influenced the low number of airborne fungal spores outside the cave and indirectly inside it, because, as mentioned above, most fungal spores in summer come from the environment surrounding the caves (Ogórek et al. 2014a, Pusz et al. 2014; Ogórek et al. 2016b, d).

We used culture-based analysis with a microbiological air sampler in our study, which is the most popular method for quantitative mycological analysis in underground sides (Ogórek and Lejman 2015). In the literature, it is reported that this method has several limitations, e.g., it can be used only for culturable microorganisms, and slow-growing colonies are difficult to detect by its use (Macher 2001). On the other hand, culture-based analysis helps explain the potential ecological roles of microorganisms in ecosystems, e.g., through the knowledge of isolates’ phenotype and their biotic properties (Boone and Castenholz 2001). Moreover, we have shown in these studies that this method is effective for detecting in the air even the spores of slow-growing fungi such as Basidiomycota. However, it refers only to cases in which the air contains few fungal spores, such as in our research. Moreover, the cost of this method is lower than for molecular techniques, and most air samplers are small, so they are useful for application in difficult conditions such as underground sites (Ogórek and Lejman 2015).

## Conclusions

The results of the first aero-mycological research in the Demänovská Ice Cave showed that the fungal spores inhabiting the air in this cave belong to unique species, among other things, because the airborne spores of *Alternaria abundans*, *Arthrinium kogelbergense*, *Cryptococcus curvatus*, *Discosia* sp., *Fomes fomentarius*, *Microdochium seminicola* and *Trametes* were discovered for the first time in natural and artificial underground sites. Moreover, the air samples collected from inside the cave contained a higher number of fungal species than samples collected outside it. Overall, the airborne spores of cosmopolitan fungal species belonging to the genus *Cladosporium* dominated in this study. However, in the case of the particular species *T. hirsuta*, spores were most commonly found in the indoor air samples, and the spores of *C. herbarum* in the outdoor air samples. The external air of the cave contained more culturable airborne fungal spores than the internal air, but this level of spore concentration was lower in comparison with similar underground sites, and it does not pose a threat to the health of tourists. Thus, aero-mycological monitoring of underground sites seems to be very important for their ecosystems and it may help reduce the risk of fungal infections in humans and other mammals that may arise in particular due to climate change. It was also found that the culture-based method and the impact method with the air sampler can be used to detect even difficult-to-culture fungi such as Basidiomycota genera. However, it refers only to an ecosystem in which the air contains few fungal spores, like in this study. Moreover, photographic documentation of the fungal phenotypes found in the Demänovská Ice Cave may be useful to identify these fungi in the future.
